# Alexithymia as a Risk Factor for an Internet Addiction in Adolescents and Young Adults with Autism Spectrum Disorder

**DOI:** 10.3390/ejihpe14030044

**Published:** 2024-03-12

**Authors:** Magdalena Anna Skotalczyk, Karolina Anna Dąbrowska, Joanna Smorońska-Rypel, Krzysztof Maria Wilczyński, Małgorzata Janas-Kozik

**Affiliations:** 1 Scientific Association at the Department of Psychiatry and Psychotherapy of Developmental Age, Medical University of Silesia in Katowice, 40-055 Katowice, Poland; 2 Department of Psychiatry and Psychotherapy of Developmental Age, Medical University of Silesia in Katowice, 40-055 Katowice, Poland; 3 Paul II Child and Family Health Centre in Sosnowiec Sp. z.o.o., 41-218 Sosnowiec, Poland

**Keywords:** alexithymia, Internet addiction, autism spectrum disorders

## Abstract

The aim of the study is to investigate the association of alexithymia with Internet addiction and autism spectrum disorders among adolescents and young adults. The links between alexithymia, ASD and other mental disorders are still a largely unexplored topic in psychiatry. An intriguing question is to what extent alexithymia can be a component of the clinical picture of ASD, and to what extent it is an independent phenomenon often co-occurring with ASD. The study group consisted of young Poles aged 11 to 35 (*n* = 229), including women (*n* = 167; 73%), men (*n* = 53; 23%) and non-binary people (*n* = 9; 4%). The following questionnaires were used in the Polish validated version as screening tools and shared online: AQ (Autism Quotient), TAS-20 (Toronto Alexithymia Scale), IAT (Internet Addiction Test). Among the subjects, 15 people admitted that they had received an official diagnosis of ASD, while 26 people showed a significantly increased severity of autistic traits on the AQ questionnaire. People with ASD who also exhibit alexithymia features are certainly more prone to problematic use of the Internet. In contrast, such a risk in people with ASD without alexithymia is comparable to the general population.

## 1. Introduction

The concept of “alexithymia” first appeared in the scientific literature in 1973 and was introduced by the American psychiatrist P. Sifneos. The term was created from three Greek words: negating prefix -a, meaning lack, lexis- meaning word, thymos- meaning emotion [1]. The construct of alexithymia is now highly studied. Its role in psychopathology and neurodevelopment is not yet clear, because it is a very transversal and complex process. It cannot be classified as a specific disorder and is rather a complex and transversal phenomenon to various psychopathological and neurodevelopmental conditions. Alexithymia is not recognized as an independent nosological unit and therefore it does not appear in the new ICD-11 or DSM-5 classification as a separate entity [2].

Alexithymia is a complex process that involves both metacognitive elements (monitoring, characterization), as well as elements of narration and interpretation, as well as aspects related to somatic and proprioceptive processes. Despite its unclear nosological status, specific “diagnostic criteria” for it were developed in the literature, which include [3]:

(1) Difficulties in differentiating emotions and insufficient understanding that selected somatic sensations can be a manifestation of emotional experiences;
(2) Difficulty verbalising emotions;
(3) Limited imagination and fantasy;
(4) Thoughts focused primarily on reality combined with a very limited or complete lack of introspection.

As an additional symptom, psychosomatic symptoms are also indicated, primarily in the field of gastrointestinal disorders or chronic pain syndromes [4].

In the general population, the prevalence of alexithymia is estimated at about 10% [5], however, in the psychiatric population this percentage is significantly higher.

Alexithymia is highly represented in some personality disorders and in some neurodevelopmental conditions. Alexithymia itself is traditionally associated with episodes of depression or autism spectrum disorder (ASD). In the population of children and adolescents with ASD, its incidence may reach up to 50% [6,7].

Autism spectrum disorders are a heterogeneous group of disorders characterized by a diverse course and clinical presentation. In the newly introduced ICD-11 classification, diagnostic criteria include persistent deficits in initiating and maintaining social contacts and interactions, as well as persistent, limited, repetitive and inflexible patterns of behavior, interests and activities that are clearly unusual or excessive in relation to the age of the person and the socio-cultural context.

The onset of the disorder is usually in the developmental period, predominantly early childhood, but the characteristic symptoms may not fully manifest themselves until later, when the social demands exceed limited possibilities [8]. Although these signs are considered axial symptoms necessary for diagnosis, they are in fact broad categories that include a wide range of different behavioral phenotypes. In addition to symptoms that fall within the diagnostic criteria, a wide range of accompanying behaviors is observed in people with ASD. These factors make the actual clinical presentation of ASD very heterogeneous. Moreover, the comorbidity and strong variability of the symptomatology depending on sex further complicates the already demanding diagnostic process.

Deficits in the theory of mind occurring in the course of ASD, leading to limitations regarding social cognition, inability to decode non-verbal messages (e.g., gestures or facial expressions) and difficulties in reading metaphors [9], are sometimes associated in the literature with co-occurring alexithymia rather than strictly sense with the clinical picture of ASD [10,11,12]. This is most likely due to the presence of alexithymia which complicates difficulties in the form of theory of mind deficits in people with ASD, additionally affecting metacognitive functions, behaviors and mechanisms of emotional and behavioral regulation.

Therefore, the question arises as to how the severity of alexithymia modifies the risk of phenomena which, according to research, people with ASD are significantly more burdened in comparison with the neurotypical population.

ASD and addiction both share features of extreme interests and rigid habits, which may predispose people to the occurrence of addictions—especially behavioral addictions such as addiction to the Internet, gaming or gambling [13]. Despite the majority of precedent studies on ASD and addiction focusing on chemical dependence, the area of behavioral addiction has not yet been thoroughly studied. Kervin R et al. (2021) in his literature review points to a positive correlation between ASD and behavioral addiction. However, most of these works also included other mental disorders. Thus, at this point, the nature of the relationship between behavioral addiction and ASD remains unclear, both correlatively and causally [14].

In today’s world, the Internet has become an inseparable tool in almost every aspect of life, so it is not surprising that in some individuals it is increasingly associated with behaviors that may bring to mind “harmful use” or even addiction to the Internet [15]. In the paediatric population, this phenomenon is a growing problem. Data show that 11.9% of young people exhibit characteristics of Internet addiction [16]. Most researchers describing the criteria of Internet addiction in particular point to the lack of control over behavior, maintaining behavior despite the visible damage it causes, a strong desire and sense of compulsion to continue it alongside the loss of other types of interests and limitations of other forms of leisure activities [17,18,19]. In Young’s studies internet addiction was described as most similar to the nature of Pathological Gambling and on account of that resemblance could be defined as an impulse-control disorder not involving an intoxicant [19]. Interestingly, many studies have highlighted the co-occurrence of autism spectrum disorders and excessive Internet use [20,21,22]. This raises the question of the nature of the relationship between these individuals. On the one hand, addiction may be an expression of reduced social competences and non-verbal communication, stereotypical interests or isolation, and thus result from the axial symptoms of ASD. Hirota and Takahashi in their study showed that features of ASD were found to be a predictor of Internet addiction, which may be associated with difficulties in communication and social relationships [23]. In contrast, another study concluded that people with ASD, due to their psychological and social problems, appear to be more likely to experience problematic Internet use because they often spend a lot of time alone and the Internet allows them to explore their interests [21]. On the other hand, an intriguing hypothesis may suggest the use of the Internet as a kind of substitute mechanism compensating for deficits caused by alexithymia. Mahapatra A. concluded in her review paper that all previous studies have shown a significant positive relationship between the severity of alexithymia and Internet addiction. However, the causal direction of the relationship is not clear due to the interplay of many other factors [24].

The aim of this study was to investigate the link between alexithymia and Internet addiction and autism spectrum disorder. It’s still a largely unexplored topic in psychiatry. It is considered to what extent alexithymia may be a component of the clinical picture of ASD, and to what extent some of the features and behaviors result independently from concomitant alexithymia.

## 2. Materials and Methods

The survey was conducted in a questionnaire (Appendix A) form via social media (primarily the facebook.com website). A link to the questionnaires was shared on forums from November 2020 to February 2021 and the participants could voluntarily and anonymously take part in the study.

### 2.1. Participants

The project involved 229 people with average age equaled 23.2 years (95%CI: 22.5–24.03; SD: 5.8; min/max: 11/35), including 167 women (73% of the participants), 53 men (23% of the participants) and 9 non-binary people (4% of the participants). Among the respondents, 15 people reported that they had been diagnosed with ASD, while 26 respondents showed a significantly increased intensity of autistic traits in the AQ questionnaire. When summed up, 30 people showed at least one of the above characteristics—having a diagnosis or a significant result in the AQ questionnaire (13% of the participants). These individuals formed a group used in statistical analysis, referred to as “people with ASD features” which consisted of 20 women, 7 men and 3 non-binary people.

### 2.2. Methods

The following tools were used in the presented study:

1. Toronto Alexithymia Scale (TAS-20) [25]—A tool for measuring the severity of alexithymia traits translated and validated for the Polish population by Ścigała D. et al. (2020), in accordance with international guidelines [26]. It is a scale consisting of twenty items, evaluated on a five-point Likert scale, in which 1 means “strongly disagree” and 5 means “strongly agree”. In total, you can get from 20 to 100 points. It is possible to get from 20 to 100 points. The TAS-20 scale measures 3 dimensions of alexithymia: difficulty in identifying emotions (DIF), difficulty in verbalizing emotions (DDF) and tendency to operational thinking style (EOT). The interpretation of individual results is as follows: a score equal to or less than 51 indicates the absence of alexithymia (130 people), 52–60 very likely alexithymia (45 people), while results equal to or greater than 61 indicate alexithymia (54 people).
2. Young’s Internet Addiction Test (IAT)—A tool used to assess the degree of Internet addiction [27]. The study used the Polish version of the IAT, validated by Hawi N. et al. (2015) [28]. It is a self-report scale of twenty points, which were assessed on a five-point Likert scale from 1 (rarely) to 5 (always). By answering individual questions, it is possible to get a total of 20 to 100 points. Based on the above test, the following groups of Internet users can be distinguished: below 30 indicates the absence of Internet addiction (49 people), the total results in the range of 31–49 indicate mild addiction (125 people), 50–79 moderate addiction (54 people), while 80–100 indicates severe addiction (1 person).
3. Autism spectrum quotient (AQ)—A tool used to measure the severity and profile of features of autism spectrum disorders. The Polish version of the AQ scale was translated by Pisula et al. (2013) [29]. The AQ test consists of 50 statements assessing five different areas: social skills, change of attention, attention to detail, communication, imagination. Participants marked their answers on a four-point Likert scale, with 1 meaning “strongly disagree” and 4 “strongly agree”. The respondent was awarded 1 point for each answer suggesting an autistic trait (poor social skills, poor communication skills, attentiveness, exceptional attention to detail, poor imagination) [30]. A score greater than or equal to 30 points meant an increased intensity of ASD features (26 people) [31].
4. In addition, the respondents completed a short demographic questionnaire including questions about age, place of residence, major, field and year of study and type of activity on the Internet. In the question about online activity, the participants had a choice of social media, games, movies/series/livestreams, content creation, exploring interests, learning and other activities.

## 3. Statistical Analysis and Results

The statistical analysis was carried out using the Statistica 13 software. The significance level was ⲁ = 0.05. The following tools were used in the statistical calculations: the Kruskall–Wallis ANOVA test, the U-Mann Whitney test, Spearman’s rho correlation and multiple regression. Finally, odds ratios were calculated for dichotomous variables.

### Results

Gender differences in ASD severity, alexithymic traits and Internet addiction were assessed (measured in the AQ, TAS-20 and IAT questionnaires, respectively). The Kruskall–Wallis ANOVA tests were used for this purpose. For variables related to ASD and Internet addiction, the result was statistically insignificant (*p* > 0.05). However, it turned out to be statistically significant for the variable presenting alexithymic features—the average score in the TAS-20 questionnaire was 45.79 in men, 51.08 in women and 62.33 in non-binary subjects.

**Figure 1:**
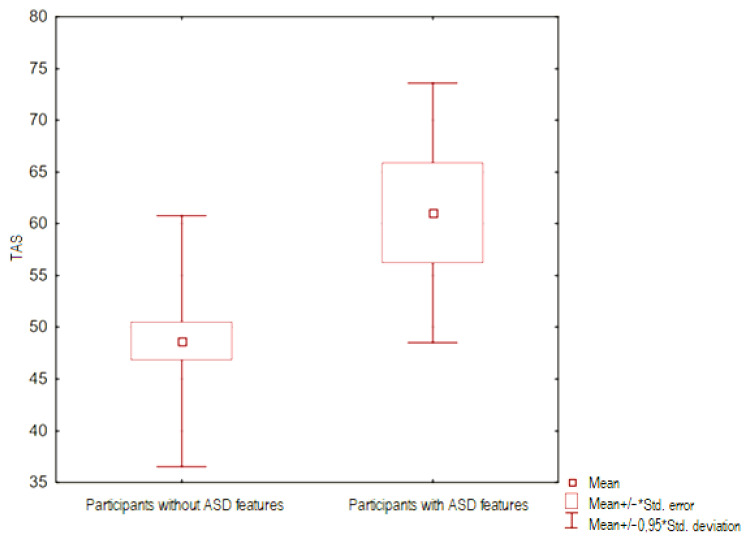
Frame-whisker plot relative to the TAS variable (intensity of alexithymic features) between groups of people with and without ASD features.

The intensity of alexithymic features (assessed using the TAS-20 questionnaire) between groups of people with and without ASD features was compared—for this purpose, the U-Mann Whitney test was performed. The result obtained was statistically significant (*p* < 0.05), it is presented in Figure 1.

**Figure 2:**
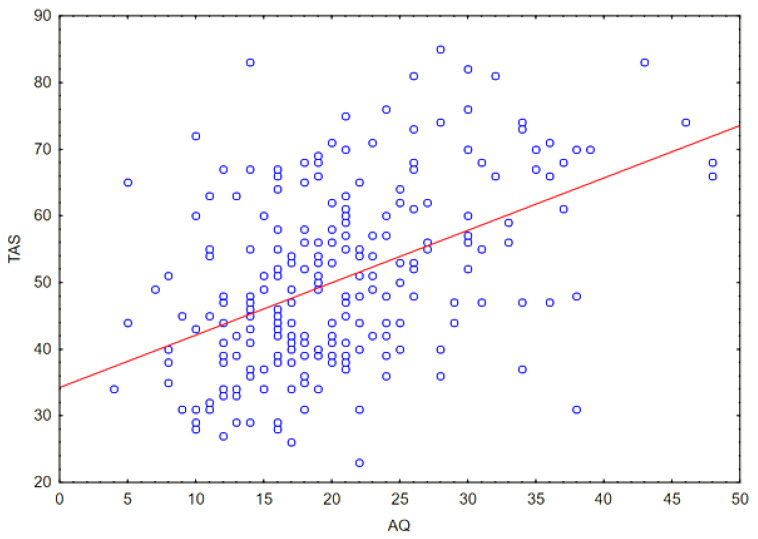
Correlation between the outcomes of TAS (intensity of alexithymic features) and AQ (intensity of ASD features).

The Spearman correlation coefficient between quantitative variables was also calculated: the TAS-20 questionnaire score and the AQ questionnaire result. A moderate correlation was obtained (*r* = 0.45; *p* < 0.05). The result is shown in Figure 2.

**Figure 3:**
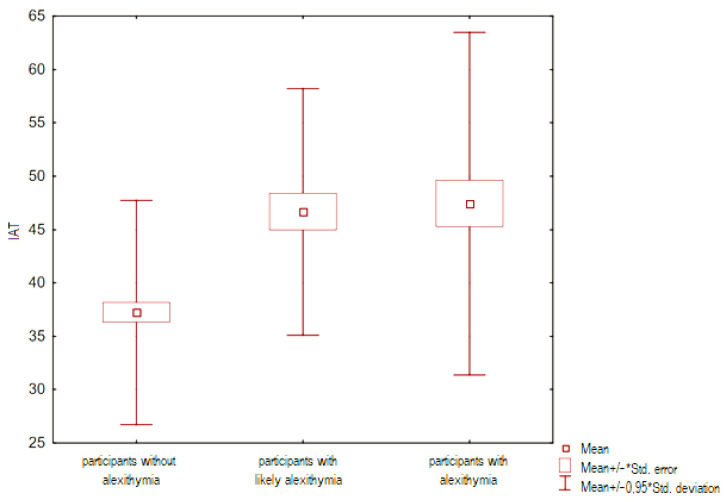
Frame-whisker graph relative to the IAT variable (level of Internet addiction) between groups of people with different intensity of alexithymia.

Next, the differences in the distribution of the quantitative variable, which was the level of Internet addiction (assessed in the IAT questionnaire), were analyzed between the groups distinguished by the TAS-20 questionnaire (the group with alexithymia, with probable alexithymia and without alexithymia were evaluated). For this purpose, the Kruskall–Wallis ANOVA test was performed. The result turned out to be statistically significant (*p* < 0.05), it is presented in Figure 3.

**Figure 4:**
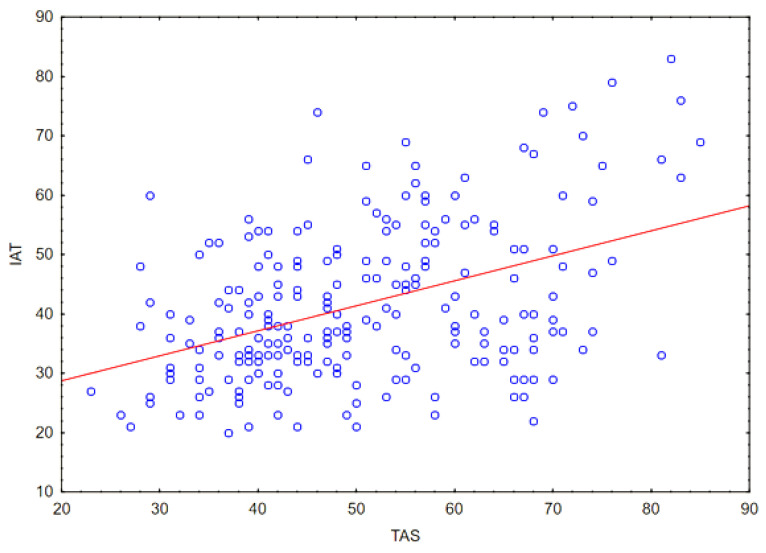
Correlation between the outcomes of IAT (level of Internet addiction) and TAS (intensity of alexithymic features).

The Spearman correlation coefficient was also calculated between quantitative variables: the level of alexithymia (assessed in the TAS-20 questionnaire) and the level of Internet addiction (assessed in the IAT questionnaire). The correlation was at a moderate level (*r* = 0.38; *p* < 0.05), the result is presented in Figure 4.

**Figure 5:**
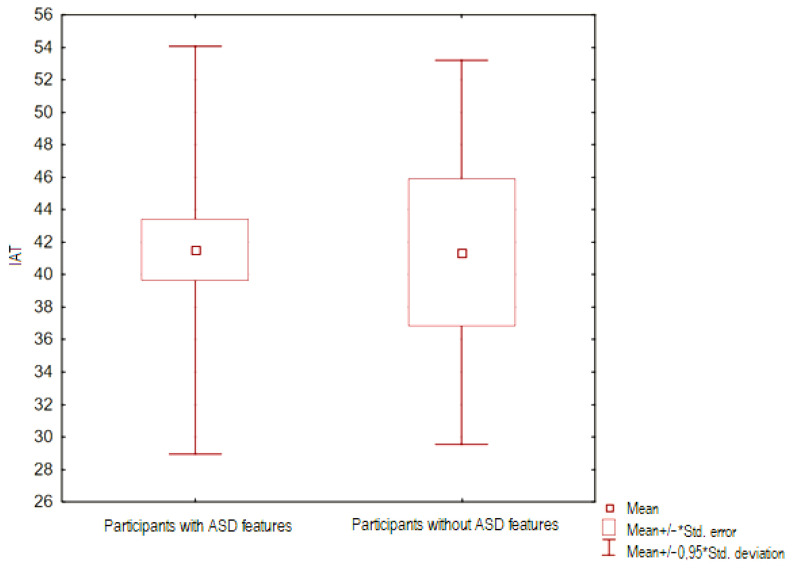
Graph of the whisker frame relative to the IAT (level of Internet addiction) variable between groups of people with and without ASD features.

The U-Mann Whitney test assessed the difference in the level of Internet addiction (assessed in the IAT questionnaire) between groups of people with and without ASD. The result obtained was statistically insignificant (*p* > 0.05), it is presented in Figure 5.

**Figure 6:**
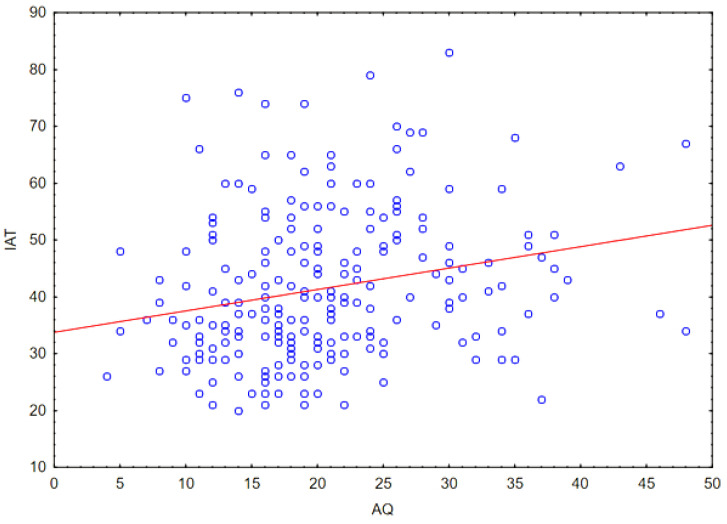
Plot of the dispersion of the IAT variable (level of Internet addiction) relative to the variable AQ (intensity of ASD features).

The correlation of the following quantitative variables was then assessed: the level of Internet addiction (assessed in the IAT questionnaire) and the level of autistic characteristics (assessed in the AQ questionnaire), for this purpose the Spearman correlation coefficient was calculated, obtaining a weak correlation (*r* = 0.27; *p* < 0.05). The result is presented in Figure 6.

Using multiple regression, the relationship between independent variables (gender, age, place of residence, education, field of study, way of spending time on the Internet, and the level of alexithymia and ASD assessed in the TAS-20 and AQ questionnaires) and the dependent variable—Internet addiction (level assessed by the IAT questionnaire) was assessed. A statistically significant result was obtained for age (a negative correlation with the level of Internet addiction was visible) and for the level of alexithymia (a positive correlation with the level of Internet addiction was visible). The results are presented in Table 1.

Odds ratios for the corresponding dichotomous variables were also calculated. The results were as follows:

- The presence of ASD features increased the risk of Internet addiction (min. 31 points of the IAT questionnaire) 0.88 times (CI: 0.35–2.19; *p* > 0.05), this result was statistically insignificant;
- The presence of ASD features increased the risk of alexithymia features (at least 52 points in the TAS-20 questionnaire) 4.36 times (CI: 1.85–10.28; *p* < 0.05), this result was statistically significant;
- The presence of alexithymia features (min. 52 points in the TAS-20 questionnaire) increased the risk of Internet addiction (min. 31 points in the IAT questionnaire) 2.74 times (95%CI: 1.34–5.58); *p* < 0.05), this result was statistically significant.

**Figure 7:**
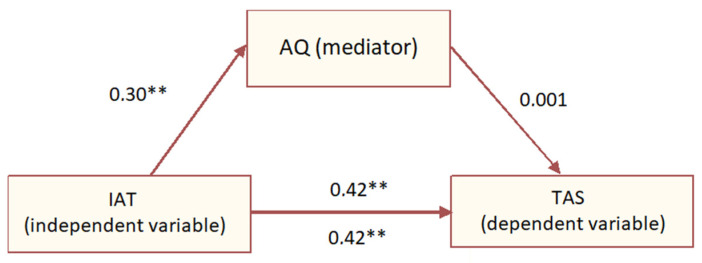
Mediation model between AQ, IAT and TAS values. The “**” punctuations imply the statistically significant values.

There was also mediational analysis done. In the first step, a regression analysis was performed between IAT and TAS variables—this model turned out to be suitable to the data (F (1, 239) = 54.13; *p* < 0.001) and explains 18% of the variance and confirms a positive relationship between the independent variable and the dependent (Beta = 0.42; *p* < 0.001). Moreover, the regression analysis between IAT and AQ variables indicates that there is a relationship between the independent variable and the mediator (Beta = 0.30; *p* < 0.001). This model is also suitable to the data (F (1, 239) = 13.04; *p* < 0.01)—however, the relationship is very weak, and the model explains only 4% of the variance. In the last step, a model was created that took into account both the role of the mediator and the independent variable, which also fits the data appropriately (F (2, 238) = 26.96; *p* < 0.001). It turned out that the role of the independent variable remained at the same level (Beta = 0.42; *p* < 0.001), and the mediator was statistically insignificant (Beta = 0.001; *p* > 0.05)—which means that it does not play a role in explaining the dependent variable. Mediation model is presented in Figure 7.

## 4. Discussion

Among the respondents to this survey, 30 people (13%) showed signs of ASD. This result differs significantly from the global ASD incidence of about 1% [32]. The apparent overrepresentation of people with autism spectrum disorders may have been due to the posting of questionnaires on social media. The forms were shared inter alia on social media profiles and in groups devoted to this disorder. It is possible that people who decided to take part in the study were interested in the above issues or may have suspected or already have been diagnosed with mental disorders. The increased use of the Internet among the population of people with ASD [21] may also partially explain this phenomenon.

In the gender distribution, a large overrepresentation of women was observed (73%) and among them ASD features were more common (66%), although this difference turned out to be statistically insignificant. This result may come as a surprise, as according to the literature males are four times more likely to be diagnosed than females [33], which is usually explained by the diagnostic difficulties linked to the different ASD presentation in females as well as inadequacy of the diagnostic tools that were designed based on the male-specific clinical presentation [34]. Furthermore, in females a phenomenon of masking symptoms of ASD due to the social training is frequently observed [35].

The overrepresentation of females in the presented study may be due to the fact that women are more likely than men to complete online surveys [36,37], while those specifically from the neuroatypical group are more likely to use or even abuse the Internet.

The lower intensity of alexithymic traits observed in our study in males also conflicts with studies suggesting a higher incidence of alexithymia in men [38], but the reason for this result can also be partly sought in the specificity of the group recruited via social media. On the other hand, it should be noted that alexithymia itself is usually associated with limited insight into one’s own problems—basically, according to literature sources, this is one of the proposed criteria symptoms of alexithymia. People with a higher severity of these types of features usually do not feel direct discomfort resulting from, for example, the emotions they experience, but rather feel it through numerous, often very intensified somatic ailments. Therefore, men as a group that would present elevated levels of alexithymia in the general population would also be the population with a reduced capacity for introspection and reporting. Therefore, the results obtained may be subject to an otherwise inevitable error leading to apparently lower levels of alexithymia in the male population.

The essence of our study was to evaluate alexithymia as a risk factor for Internet addiction in adolescents and young adults with autism spectrum disorder. Outcomes of the TAS-20 questionnaire used in our research showed that as many as 24% of respondents present significant features of alexithymia, while in the literature its prevalence is estimated at about 10% of the general population [5].

The results of the study show a higher incidence of alexithymic features in people with autistic features. The ASD population reported significantly higher total alexithymia scores than neurotypical subjects (they scored significantly higher on the TAS-20 subscales). This corresponds with the conclusions presented in previous works [39]. Drawing attention to the uncertain nosological status of alexithymia, which is described in the literature as a personality trait or a set of symptoms, or even a disorder, the question of the relationship between it and ASD is intriguing. Symptoms characteristic of alexithymia [3], such as poor insight into emotions, difficulties with verbalizing them, primarily on the basis of non-verbal communication disorders, limited imagination and the ability to fantasize are also traditionally associated with the clinical picture of ASD. For this reason, an intriguing topic of consideration is the question of whether alexithymia is an element of the clinical picture of ASD resulting directly from its axial symptoms, or rather it is a co-occurring phenomenon independent of the clinical presentation of autistic features. Why is this issue important? Well, hypothetically, if we assume that alexithymia is an independent phenomenon, co-occurring with ASD in a significant percentage of patients, the question arises as to what extent it modifies the course and clinical picture, as well as susceptibility, for example, to certain forms of treatment proposed in scientific studies. An example of this is the use of intranasal supply of oxytocin. This treatment is currently being intensively studied in the ASD population for alleviating the symptoms of social cognitive impairment. Interestingly, the results obtained, although optimistic, are often contradictory, and the evidence for its effectiveness is currently assessed as weak [40]. However, taking into account the effectiveness of this method also in the neurotypical population of people with social difficulties, as well as the significant impact of alexithymia on its effects [41], it seems important to ask to what extent the results of studies on the supply of oxytocin in patients with ASD are not distorted by the co-occurrence of alexithymia. The alternative hypothesis, i.e., alexithymia as an element of the clinical picture of ASD, raises again the question of the risk of underdiagnosed ASD in apparently neurotypical individuals with elevated alexithymic features. Of course, these considerations are beyond the scope of interest of the presented study and require in-depth research to obtain an answer to these questions.

The above statistical analysis also showed that the risk of Internet addiction in the group of people with high levels of alexithymia is significantly increased. However, in the group of people with ASD, this risk was not significantly higher compared to the neurotypical population. Interestingly, conclusions drawn in another study showed a higher prevalence of excessive internet use among participants with ASD (at the level of 10.8%). It should be noted, however, that in their analysis they did not take into account the aspect of alexithymia [42]. Many studies to date have shown a significant positive correlation between the presence of alexithymia and the severity of Internet addiction. However, the causal direction of the relationship is not clear due to the interplay of many other factors [24]. Our analysis confirms the positive correlation of these phenomena. In our study, the assessment of ASD and alexithymia as risk factors for Internet addiction showed that people with detected alexithymia were more likely to present symptoms of Internet addiction. In contrast, in people with ASD, this risk is comparable to neurotypical people, and the poor correlation of these phenomena is most likely due to the high incidence of alexithymia in these persons. Given that alexithymia is a condition in which the patient is “blind” to their emotional states and signals from inside the body and tends to confuse them, these people will hypothetically have impaired decision-making processes and a limited ability to defer reward. These mechanisms are based on the balance between the emotional-visceral component and the rational component. However, in order to effectively modulate the activity of the emotional-visceral component, it is necessary to be able to perceive and interpret its signals. High levels of alexithymia hypothetically prevent this by significantly distorting the decision-making processes associated with deferring gratification [43]. For this reason, alexithymic people may have a tendency to seek quick and easy sources of gratification, such as using the Internet, and may also be more predisposed to addiction. This hypothesis seems to be consistent with the results obtained and provides an interesting point of view on this issue. The relationship between alexithymia, the ability to postpone gratification and addiction seems to be an interesting topic and the direction in which further research in this field may go.

Another variable in the population that correlated with internet addiction in our analysis was age. This was an inversely proportional relationship, with young people showing a greater propensity for problematic use of the Internet (96% of people under 18 years of age presented a minimum mild addiction in the IAT questionnaire). Studies indicate Internet addiction as a condition to which young people are extremely susceptible. It can also be associated with other mental health difficulties, such as depression, anxiety disorders or sleep problems [44].

Although the presented results seem reliable, it should be remembered that the presented study has some limitations. They primarily include the form of data acquisition, i.e., self-assessment questionnaire disseminated through the social media. These types of research are burdened with the risk of underestimated results resulting from difficulties in negative assessment of one’s own behavior. Secondly, clinical diagnosis cannot be made on the basis of the results obtained in the questionnaire study. Presented results are only estimations of the severity of the characteristics of a given disorder in the studied population. Moreover, in the context of both autistic and alexithymia traits, it should be remembered that these traits are likely to be associated with limited insight into one’s own difficulties. For this reason, the methodology of the survey, based on self-report questionnaires, may be burdened with error in the form of underestimating the results obtained. However, this type of error in the context of the fundamental question of the relationship between Internet addiction and alexithymia is likely to reduce the statistical significance of the results rather than the risk of misassuming the alternative hypothesis (about the existence of a relationship). The last issue is the age of the respondents included in the presented survey. It should be taken into account that age may hypothetically have a significant impact on internet usage habits in the general population. Therefore, it is important to take this into account when interpreting the presented results. However, on the other hand, it should be remembered that the specificity of the studied population—people who actively use social media and spend their free time there—means that regardless of the age group, it is a selected population and more homogeneous in terms of habits related to the use of the Internet than the general population. Therefore, the age effect should be significantly smaller than expected for the general population.

## 5. Conclusions

Based on the above analysis, it can be concluded that alexithymia significantly modifies the risk of Internet addiction. People with ASD who also show alexithymia features are definitely more exposed to problematic use of the Internet. In contrast, such a risk in people with ASD without alexithymia is comparable to the general population. It is worth considering this issue, especially in clinical practice. Studying alexithymic features in people with ASD, rather than the diagnosis itself, could help identify people who are particularly vulnerable to Internet addiction.

In the context of understanding the presented results, as well as future directions of research on this issue, the topic of the ability of people with alexithymia to introspect and report their own difficulties seems to be very interesting. This is an issue that requires methodologically thoughtful research, including, for example, third-party reporting. Hypothetically, there is a risk that the actual level of alexithymia could be non-linearly correlated with the results of self-report TAS-20 tests. Up to a certain point, the relationship could be linear, and above that, it could be a negative correlation. This would have a significant negative impact on the results of existing research, significantly limiting the ability to reliably interpret the results obtained.

Another issue is the relationship between the reward system, decision-making processes and alexithymia. Explaining the observed relationship between Internet addiction and alexithymia with the hypothesis that this condition interferes with the ability to postpone decisions, it seems interesting to conduct further research, firstly, on the nature of this relationship and, secondly, on the general risk of addiction in this context.

## Figures and Tables

**Table 1:** Multiple regression relative to the IAT (level of Internet addiction) variable.

N = 229	b *	Std. Error from b *	b	t (220)	t (220)	*p*
Absolute term			39.562	6.785	5.831	0.000
Gender	−0.060	0.065	−1.442	1.570	−0.918	0.359
Age	−0.038	0.062	−0.564	0.210	−2.680	0.008
Place of residence	−0.038	0.062	−0.434	0.703	−0.618	0.537
Education	−0.100	0.061	−0.014	0.009	−1.629	0.105
Field of study	0.042	0.066	0.001	0.002	0.644	0.520
Way of spending time on the internet	−0.115	0.062	−0.782	0.421	−1.860	0.064
Level of alexithymia	0.338	0.071	0.320	0.069	4.762	0.000
Level of ASD	0.090	0.069	0.147	0.112	1.310	0.191

## Data Availability

The data presented in this study are available on request from the corresponding author.

## Appendix A

Self-report demographic questionnaire went as following:

1. Select your gender.

1.1. Male

1.2. Female

1.3. Other (Enter your response)

2. Enter age (in years).

3. Select your place of residence:

4.1. Countryside

4.2. City (less than 100,000 residents)

4.3. City (100,000–300,000 residents)

4.4. City (more than 300,000 residents)

5. Select your level of education.

5.1. In primary school

5.2. In high school/technical school

5.3. During studies

5.4. Primary education

5.5. Secondary education.

5.6. Higher education

6. Select your field of study (unless not applicable).

6.1. Humanities

6.2. Engineering and technology

6.3 Medical and health sciences

6.4. Agriculture sciences

6.5. Social sciences

6.6. Natural sciences

6.7. Theology

6.8. The arts

7. Enter your major (unless not applicable).

8. Select your year of study (unless not applicable).

8.1. 1st

8.2. 2nd

8.3. 3rd

8.4. 4th

8.5. 5th

8.6. 6th

8.7. During studies.

9. Select online activity the most often chosen by you.

9.1. Social media

9.2. Games

9.3. Movies/series/livestreams

9.4. Content creation

9.5. Exploring interest

9.6. Learning

9.7. Other activities.

Following polish versions of questionnaires were used:

I. Toronto Alexithymia Scale (TAS-20)—questionnaire of 20 points assessed on a scale from 1 to 5

(1) Strongly Disagree

(2) Disagree

(3) Neither Agree nor Disagree

(4) Agree

(5) Strongly Agree

1. I am often confused about what emotion I am feeling.

2. It is difficult for me to find the right words for my feelings.

3. I have physical sensations that even doctors don’t understand.

4. I am able to describe my feelings easily.

5. I prefer to analyze problems rather than just describe them.

6. When I am upset, I don’t know if I am sad, frightened, or angry.

7. I am often puzzled by sensations in my body.

8. I prefer to just let things happen rather than to understand why they turned out that way.

9. I have feelings that I can’t quite identify.

10. Being in touch with emotions is essential.

11. I find it hard to describe how I feel about people.

12. People tell me to describe my feelings more.

13. I don’t know what’s going on inside me.

14. I often don’t know why I am angry.

15. I prefer talking to people about their daily activities rather than their feelings.

16. I prefer to watch “light” entertainment shows rather than psychological dramas.

17. It is difficult for me to reveal my innermost feelings, even to close friends.

18. I can feel close to someone, even in moments of silence.

19. I find examination of my feelings useful in solving personal problems.

20. I look for hidden meanings in movies or plays.

II. Young’s Internet Addiction Test (IAT)—a questionnaire of 20 points assessed on a scale from 1 to 5:

(1) Rarely

(2) Occasionally

(3) Frequently

(4) Often

(5) Always

1. How often do you find that you stay online longer than you intended?

2. How often do you neglect household chores to spend more time online?

3. How often do you prefer the excitement of the Internet to intimacy with your partner?

4. How often do you form new relationships with fellow online users?

5. How often do others in your life complain to you about the amount of time you spend online?

6. How often do your grades or school work suffer because of the amount of time you spend online?

7. How often do you check your email before something else that you need to do?

8. How often does your job performance or productivity suffer because of the Internet?

9. How often do you become defensive or secretive when anyone asks you what you do online?

10. How often do you block out disturbing thoughts about your life with soothing thoughts of the Internet?

11. How often do you find yourself anticipating when you will go online again?

12. How often do you fear that life without the Internet would be boring, empty, and joyless?

13. How often do you snap, yell, or act annoyed if someone bothers you while you are online?

14. How often do you lose sleep due to being online?

15. How often do you feel preoccupied with the Internet when off-line, or fantasize about being online?

16. How often do you find yourself saying “just a few more minutes” when online?

17. How often do you try to cut down the amount of time you spend online and fail?

18. How often do you try to hide how long you’ve been online?

19. How often do you choose to spend more time online over going out with others?

20. How often do you feel depressed, moody, or nervous when you are off-line, which goes away once you are back online?

III. Autism spectrum quotient (AQ)—a questionnaire of 50 points assessed on a scale from 1 to 4:

(1) Strongly Disagree

(2) Slightly Disagree

(3) Slightly Agree

(4) Strongly Agree

1. I prefer to do things with others rather than on my own.

2. I prefer to do things the same way over and over again.

3. If I try to imagine something, I find it very easy to create a picture in my mind.

4. I frequently get so strongly absorbed in one thing that I lose sight of other things.

5. I often notice small sounds when others do not.

6. I usually notice car number plates or similar strings of information.

7. Other people frequently tell me that what I’ve said is impolite, even though I think it is polite.

8. When I’m reading a story, I can easily imagine what the characters might look like.

9. I am fascinated by dates.

10. In a social group, I can easily keep track of several different people’s conversations.

11. I find social situations easy.

12. I tend to notice details that others do not.

13. I would rather go to a library than to a party.

14. I find making up stories easy.

15. I find myself drawn more strongly to people than to things.

16. I tend to have very strong interests, which I get upset about if I can’t pursue.

17. I enjoy social chitchat.

18. When I talk, it isn’t always easy for others to get a word in edgewise.

19. I am fascinated by numbers.

20. When I’m reading a story, I find it difficult to work out the characters’ intentions.

21. I don’t particularly enjoy reading fiction.

22. I find it hard to make new friends.

23. I notice patterns in things all the time.

24. I would rather go to the theater than to a museum.

25. It does not upset me if my daily routine is disturbed.

26. I frequently find that I don’t know how to keep a conversation going.

27. I find it easy to “read between the lines” when someone is talking to me.

28. I usually concentrate more on the whole picture, rather than on the small details.

29. I am not very good at remembering phone numbers.

30. I don’t usually notice small changes in a situation or a person’s appearance.

31. I know how to tell if someone listening to me is getting bored.

32. I find it easy to do more than one thing at once.

33. When I talk on the phone, I’m not sure when it’s my turn to speak.

34. I enjoy doing things spontaneously.

35. I am often the last to understand the point of a joke.

36. I find it easy to work out what someone is thinking or feeling just by looking at their face.

37. If there is an interruption, I can switch back to what I was doing very quickly.

38. I am good at social chitchat.

39. People often tell me that I keep going on and on about the same thing.

40. When I was young, I used to enjoy playing games involving pretending with other children.

41. I like to collect information about categories of things (e.g., types of cars, birds, trains, plants).

42. I find it difficult to imagine what it would be like to be someone else.

43. I like to carefully plan any activities I participate in.

44. I enjoy social occasions.

45. I find it difficult to work out people’s intentions.

46. New situations make me anxious.

47. I enjoy meeting new people.

48. I am a good diplomat.

49. I am not very good at remembering people’s date of birth.

50. I find it very easy to play games with children that involve pretending.

## References

[1] Maruszewski T., Ścigała E. (1998). Emotions–Alexithymia–Cognition.

[2] Halicka M., Herzog-Krzywoszańska R. (2016). Unfathomable emotions—Alexithymia from a neuropsychological perspective. Neuropsychiatr. Neuropsychol..

[3] Kooiman C.G., Spinhoven P., Trijsburg R.W. (2002). The assessment of alexithymia: A critical review of the literature and a psychometric study of the Toronto Alexithymia Scale-20. J. Psychosom. Res..

[4] Larsen J.K., Brand N., Bermond B., Hijman R. (2003). Cognitive and emotional characteristics of alexithymia: A review of neurobiological studies. J. Psychosom. Res..

[5] Hemming L., Haddock G., Shaw J., Pratt D. (2019). Alexithymia and its associations with depression, suicidality, and aggression: An overview of the literature. Front. Psychiatry.

[6] Berthoz S., Hill E.L. (2005). The validity of using self-reports to assess emotion regulation abilities in adults with autism spectrum disorder. Eur. Psychiatry.

[7] Poquérusse J., Pastore L., Dellantonio S., Esposito G. (2018). Corrigendum: Alexithymia and Autism Spectrum Disorder:A Complex Relationship. Front. Psychol..

[8] Doernberg E., Hollander E. (2016). Neurodevelopmental Disorders (ASD and ADHD): DSM-5, ICD-10, and ICD-11. CNS Spectrums.

[9] Trambacz-Oleszak S., Nosulia T. (2021). Genetic factors in Autism Spectrum Disorders (ASD). Progress Biochem..

[10] Siegal M., Varley R. (2002). Neural systems involved in “theory of mind”. Nat. Rev. Neurosci..

[11] Bird G., Cook R. (2013). Mixed emotions: The contribution of alexithymia to the emotional symptoms of autism. Transl. Psychiatry.

[12] Brewer R., Happé F., Cook R., Bird G. (2015). Commentary on “Autism, oxytocin and interoception”: Alexithymia, not Autism Spectrum disorders, is the consequence of interoceptive failure. Neurosci. Biobehav. Rev..

[13] Wijngaarden-Cremers P., Gaag R. (2015). Co-Occurring Addictive and Psychiatric Disorders: A Practice-Based Handbook from a European Perspective.

[14] Kervin R., Berger C., Moon S.J., Hill H., Park D., Kim J.W. (2021). Behavioral addiction and autism spectrum disorder: A systematic review. Res. Dev. Disabil..

[15] Bird G., Silani G., Brindley R., White S., Frith U., Singer T. (2010). Empathic brain responses in insula are modulated by levels of alexithymia but not autism. Brain.

[16] Makaruk K., Włodarczyk J., Skoneczna P. (2019). Problematic Internet Use by Youth—Research Report.

[17] Augustynek A. (2010). Computer Addictions. Diagnosis, Prevalence, Therapy.

[18] Woronowicz B.T., Woronowicz B.T. (2009). Computer and Internet addictions. Addiction. Genesis, Therapy, Recovery.

[19] Young K.S. (1998). Internet addiction: The emergence of a new clinical disorder. CyberPsychology Behav..

[20] Restrepo A., Scheininger T., Clucas J., Alexander L., Salum G.A., Georgiades K., Paksarian D., Merikangas K.R., Milham M.P. (2020). Problematic internet use in children and adolescents: Associations with psychiatric disorders and impairment. BMC Psychiatry.

[21] Coskun M., Hajdini A., Alnak A., Karayagmurlu A. (2020). Internet Use Habits, Parental Control and Psychiatric Comorbidity in Young Subjects with Asperger Syndrome. J. Autism Dev. Disord..

[22] Pluhar E., Kavanaugh J.R., A Levinson J., Rich M. (2019). Problematic interactive media use in teens: Comorbidities, assessment, and treatment. Psychol. Res. Behav. Manag..

[23] Hirota T., Takahashi M., Adachi M., Sakamoto Y., Nakamura K. (2021). Neurodevelopmental Traits and Longitudinal Transition Patterns in Internet Addiction: A 2-year Prospective Study. J. Autism Dev. Disord..

[24] Mahapatra A., Sharma P. (2018). Association of Internet addiction and alexithymia—A scoping review. Addict. Behav..

[25] Bagby R.M., Parker J.D.A., Taylor G.J. (1994). The twenty-item Toronto Alexithymia scale—I. Item selection and cross-validation of the factor structure. J. Psychosom. Res..

[26] Ścigała D.K., Zdankiewicz-Ścigała E., Bedyńska S., Kokoszka A. (2020). Psychometric Properties and Configural Invariance of the Polish—Language Version of the 20-Item Toronto Alexithymia Scale in Non-clinical and Alcohol Addict Persons. Front. Psychol..

[27] Young K.S., Van de Creek L., Jackson C. (1999). Internet addiction: Symptoms, evaluation and treatment. Innovations in Clinical Practice: A Source Book.

[28] Hawi N.S., Blachnio A., Przepiorka A. (2015). Polish validation of the internet addiction test. Comput. Hum. Behav..

[29] Pisula E., Kawa R., Shostakevich Ł., Lutsk I., Kawa M., Rynkiewicz A. (2013). Autistic traits in male and female students and individuals with high functioning autism spectrum disorders measured by the Polish version of the autism-spectrum quotient. PLoS ONE.

[30] Baron-Cohen S., Wheelwright S., Skinner R., Martin J., Clubley E. (2001). The Autism-Spectrum Quotient (AQ): Evidence from Asperger Syndrome/High-Functioning Autism, Males and Females, Scientists and Mathematicians. J. Autism Dev. Disord..

[31] Baron-Cohen S., Hoekstra R.A., Knickmeyer R., Wheelwright S. (2006). The Autism-Spectrum Quotient (AQ)—Adolescent version. J. Autism Dev. Disord..

[32] Lotufo Denucci B., Silva de Lima L., Ferreira Lima Mota I., Rocha Madureira Azevedo J., Germino Veras L., Montenegro Luzardo Bicca J.V., de Miranda Santana B., Beserra Pinheiro G., Gonçalves Coelho G., Mortari M.R. (2021). Current knowledge, challenges, new perspectives of the study, and treatments of Autism Spectrum Disorder. Reprod. Toxicol..

[33] Williams O.O.F., Coppolino M., Perreault M.L. (2021). Sex differences in neuronal systems function and behaviour: Beyond a single diagnosis in autism spectrum disorders. Transl. Psychiatry.

[34] Beggiato A., Peyre H., Maruani A., Scheid I., Rastam M., Amsellem F., Gillberg C.I., Leboyer M., Bourgeron T., Gillberg C. (2017). Gender differences in autism spectrum disorders: Divergence among specific core symptoms. Autism Res..

[35] Green R.M., Travers A.M., Howe Y., McDougle C.J. (2019). Women and Autism Spectrum Disorder: Diagnosis and Implications for Treatment of Adolescents and Adults. Curr. Psychiatry Rep..

[36] Długosz P. (2020). Report from the Second Stage of Research of UP Students. Opinion on Remote Learning and Mental Well-Being.

[37] Cat M. Conference Poll Polish. Method, Ethics, Media. Guarding the Polls. http://nastrazysondazy.uw.edu.pl/konferencja2/M_Kot.pdf?fbclid=IwAR2Y7fUAjIQHbmzkDW5N03-rj1sk3xU2BO1RjK3cJFURUlFPkjhmshceGS0.

[38] Levant R.F., Hall R.J., Williams C.M., Hasan N.T. (2009). Sex differences in alexithymia: A review. Psychol. Men Masculinities.

[39] Oakley B.F.M., Jones E.J.H., Crawley D., Charman T., Buitelaar J., Tillmann J., Murphy D.G., Loth E., EU-AIMS LEAP Group (2022). Alexithymia in autism: Cross-sectional and longitudinal associations with social-communication difficulties, anxiety and depression symptoms. Psychol. Med..

[40] Huang Y., Huang X., Ebstein R.P., Yu R. (2021). Intranasal oxytocin in the treatment of autism spectrum disorders: A multilevel meta-analysis. Neurosci. Biobehav. Rev..

[41] Luminet O., Grynberg D., Ruzette N., Mikolajczak M. (2011). Personality-dependent effects of oxytocin: Greater social benefits for high alexithymia scorers. Biol. Psychol..

[42] So R., Makino K., Fujiwara M., Hirota T., Ohcho K., Ikeda S., Tsubouchi S., Inagaki M. (2017). The Prevalence of Internet Addiction Among a Japanese Adolescent Psychiatric Clinic Sample with Autism Spectrum Disorder and/or Attention-Deficit Hyperactivity Disorder: A Cross-Sectional Study. J. Autism Dev. Disord..

[43] Scarpazza C., Sellitto M., di Pellegrino G. (2017). Now or not-now? The influence of alexithymia on intertemporal decision-making. Brain Cogn..

[44] Marin M.G., Nuñez X., de Almeida R.M.M. (2021). Internet Addiction and Attention in Adolescents: A Systematic Review. Cyberpsychol. Behav. Soc. Netw..

